# Prospective applications of bioactive materials in orthopedic therapies: A review

**DOI:** 10.1016/j.heliyon.2024.e36152

**Published:** 2024-08-10

**Authors:** Wenqing Liang, Chao Zhou, Juqin Bai, Hongwei Zhang, Hengguo Long, Bo Jiang, Jiangwei Wang, Xiaogang Huang, Hengjian Zhang, Jiayi Zhao

**Affiliations:** a Department of Orthopaedics, Zhoushan Hospital of Traditional Chinese Medicine Affiliated to Zhejiang Chinese Medical University, Zhoushan, 316000, China; b Department of Orthopedics, Zhoushan Guanghua Hospital, Zhoushan, 316000, China; c Rehabilitation Department, Zhoushan Hospital of Traditional Chinese Medicine Affiliated to Zhejiang Chinese Medical University, Zhoushan, 316000, China; d Medical Research Center, Zhoushan Hospital of Traditional Chinese Medicine Affiliated to Zhejiang Chinese Medical University, Zhoushan, 316000, China

**Keywords:** Bioactive materials, Orthopedic therapies, Ceramic nanomaterials, Polymer composites

## Abstract

The biomedical application of biodegradable polymers for addressing bone-related diseases has garnered considerable attention in recent years. Advances in material technology have expanded the repertoire of materials suitable for orthopedic implants, with nanomaterials playing a pivotal role in replicating crucial surface properties akin to natural tissues. This comprehensive review explores the evaluation of bioactive glass ceramics, shedding light on their properties and applications. The synthesis of composites through composite manufacturing has emerged as a strategy to enhance biocompatibility and biomechanical characteristics. They are addressing challenges associated with conventional implants and nanomaterials, whether in the form of functional nano coatings or nanostructured surfaces, present opportunities to refine implant techniques. Novel developments in orthopedic biomaterials, such as smart biomaterials, porous structures, and 3D implants, offer stimuli-responsive behavior to achieve desired implant shapes and characteristics. Bioactive and biodegradable porous polymer/inorganic composite materials are explored for bone tissue engineering scaffolds, aiming to promote bone formation and regeneration. As a prospective direction, the integration of stem cells into scaffolds hints at the creation of next-generation synthetic/living hybrid biomaterials, displaying high adaptability in biological settings. This review establishes a foundation for nanotechnology-driven biomaterials by elucidating fundamental design factors crucial for orthopedic implant performance and their response to cell differentiation, proliferation, and adhesion.

## Graphical abstract

**Figure:**
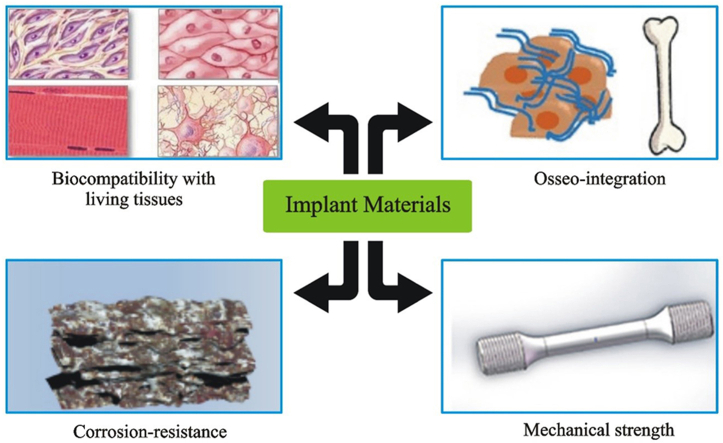

## 1 Introduction

Orthopedic therapies have witnessed remarkable advancements in recent years, shaping the landscape of patient care and outcomes. Emerging technologies and novel therapeutic strategies have redefined the treatment paradigm, emphasizing precision and patient-centric approaches [1]. Improved current studies have highlighted the integration of advanced materials, such as bioactive nanomaterials and smart biomaterials into orthopedic implants, promoting improved osteointegration and enhanced bone regeneration [2,3]. The quest for more effective drug delivery systems has led to innovations that optimize therapeutic concentrations locally, mitigating systemic side effects.

In recent years, bioactive materials have emerged as integral components in the dynamic landscape of orthopedics, presenting a transformative paradigm for therapeutic interventions. These materials, exemplified by renowned substances (e.g., hydroxyapatite, ceramics, etc), are characterized by a unique set of properties that render them highly conducive to orthopedic applications. Their composition, often tailored to mimic the natural environment of bone tissues, includes (e.g., calcium phosphate, etc). Their notable properties such as biocompatibility and osteoconductivity, position them as ideal candidates for addressing the complex challenges inherent in musculoskeletal disorders. The structures and characteristics of materials can be changed at the nanometer scale using biological, chemical, and physical processes in the multidisciplinary field of nanotechnology. Nanomaterials display novel size-dependent properties in opposition to their largest counterparts. Advances in nanotechnology have disclosed a wide range of further prospects for uses in the areas of medicine [4], environmental science [5], molecular biology [6], and biotechnology [7]. The development of numerous sophisticated methods for preventing, diagnosing, and treating numerous diseases involving medical imaging, cancer therapy, drug delivery, immunotherapy scaffolds, and tissue engineering has made it easier to realize the potential uses of nanotechnology in medicine (such as nanomedicine).

Within the field of orthopedics, the applications of these bioactive materials cover a broad spectrum. One notable application is in bone regeneration, where these materials play a pivotal role in facilitating and accelerating the natural healing processes. Their ability to integrate seamlessly with the biological milieu promotes cellular adhesion and proliferation, fostering an environment conducive to effective tissue regeneration. Furthermore, bioactive materials have proven instrumental in enhancing the performance of orthopedic implants. Their incorporation into implant structures enhances osseointegration, mitigating the risks of implant loosening and promoting long-term stability [8]. Nanomaterials are exceptionally promising biomaterials for the development of future orthopedic implants because they can imitate or recreate the individual organs of a natural bone [9]. The use of bone substitutes is essential in orthopedic uses to treat irreparable damage to healthy and natural bone. Nanomaterials can be very helpful in this regard by controlling cell growth, differentiation, and migration in addition to offering cell structural support (such as nonfunctionalized scaffolds) [10]. According to reports, the number of publications mentioning "nanotechnology for implants" quickly grows each year.

Bone related diseases are becoming one of the major clinical health concerns worldwide, particularly among the older age group people. Different kinds of biomaterials and devices have been extensively used to heal and repair damaged skeletal system parts with broken hard tissues. Metals and ceramics are examples of inorganic materials, and biological materials are another (polymers). There is a potential limitation wherein single-class materials may not fully satisfy the requirements of a particular implant application. Therefore, mixtures with multiscale designs and required properties for uses are possible by combining two or more classes. Cobalt-chromium (CoCr) alloys, stainless steels, and titanium (Ti), alongside its alloys, among others, serve a significant part as biomaterials in the substitution or fixation of damaged load-bearing bones. Metallic biomaterials, known for their high fracture hardness, flexibility, and strength, comprise over seventy percent of implant devices [11]. Despite having high mechanical strength, metallic biomaterials still have a problem with biomechanical compatibility because of their higher elastic modulus than natural bone.

The demand for bioimplants is increasing exponentially as the population matures, lifestyles shift (particularly those contributing to chronic diseases like osteoarthritis and cardiovascular diseases), bioengineering technology advances, and consumer awareness of cosmetic implants grows. Market research reports predict that the global bioimplant market will reach $115.8 billion by 2020, growing at a compound annual growth rate (CAGR) of 10.4 % from 2014 to 2020 [12]. Bioimplants have become a hopeful treatment option for a variety of conditions, including dental disorders, disfigurement, orthopedic problems, cardiovascular illness, visual impairments, neurological disorders, and other disorders [13]. Intraocular lenses, dental implants, ligaments, heart valves, sutures, bone plates, vascular grafts, joint replacements, and many other bioimplants are frequently used to (i) aid in healing, (ii) correct irregularities for cosmetic reasons, (iii) substitute or restore the function of cells that have been damaged or destroyed, and (iv) alter a bodily part's capabilities [14].

The nanotechnology influence on the implant area has started to increase over the last few years significantly. Researchers are particularly motivated by nanomaterials with biologically inspired characteristics to investigate their role in enhancing the efficacy of conventional implants. To provide a comprehensive overview of this quickly developing scientific area, the most recent advancements in crucial orthopedic biomaterials— smart biomaterials, porous materials, and 3D printed nano-composite implants—as well as the practical challenges, are addressed. This research establishes a strong base for the placement of nanotechnology-powered musculoskeletal devices inside the human body. This review aims to comprehensively explore the current landscape of bioactive materials in orthopedics, it will highlight their diverse applications and the scientific principles behind their effectiveness by synthesizing and critically analyzing the existing literature.

## 2 Orthopedic biomaterials: overview

Biomaterials for orthopedics are substances made to strengthen, replace, or sustain broken bones and tissues. Their ability to repair bones, repair joints and regenerate tissue is vital to orthopedic surgery. The effectiveness of these materials depends on their biocompatibility, mechanical properties and ability to integrate with the host tissue [15]. Recent orthopedic biomaterials research is concentrating on improving implant biocompatibility and functionality. Nanomaterials are the product of advances in nanotechnology mimic the natural bone environment promoting improved osseointegration and regeneration. Adaptive biomaterials that respond to environmental stimuli, such as PH or temperature changes, are also gaining attention for their potential to improve the performance of orthopedic implants [16]. Emerging technologies in orthopedic biomaterials include 3D printing and bio fabrication that enable the development of personalized implants customized to each patient's unique anatomy. Integrating stem cells with biomaterials is another promising area aiming to develop hybrid materials that support tissue regeneration and repair. Furthermore, bioactive glass and ceramic use is being investigated for their capacity to stimulate bone formation and enhance implant integration [17].

## 3 Orthopedic biomaterials: classification and related difficulties

The two primary divisions of biological tissues are hard tissues, such as cartilage, nails, teeth, and bone, second soft tissues, such as tendons, fibrous tissues, epidermis, and synovial membranes. The shortage of donor organs has prompted the researchers to develop novel approaches to mimic or reproduce these tissues [18]. Bioimplants have been developed to meet this demand by restoring, supporting, or enhancing the functions of human cells. Unlike biological materials like bone and tissues, biomaterials are designed for implant applications. These biomaterials, which can be synthetic or native, are intended to work accurately in a biological setting.

**Fig. 1:**
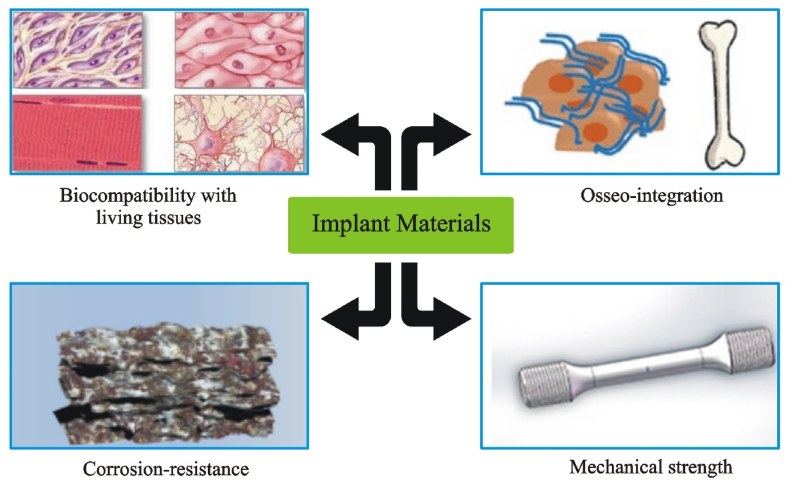
The biocompatibility of orthopedic biomaterials with living tissues, mechanical characteristics, osseointegration, and corrosion resistance are important design considerations. (such as surface roughness, hardness, strength, and flexibility).

In orthopedic applications, biomaterials are intended to either substitute or repair the structural integrity of fractured bone. Each biomaterial must fulfill some important criteria when being designed, including the right mechanical properties (such as elastic modulus and specific weight), effective bio-stability (hydrolysis, resistance to corrosion, and oxidation), osseointegration (for bone prosthetics), high wear resistance, biocompatibility, high bio-inertness (non-irritant, and non-toxic), and ease of surgical procedures (Fig. 1) [19]. Biomaterials have demonstrated effectiveness in tissue remodeling and cell growth [20].

**Fig. 2:**
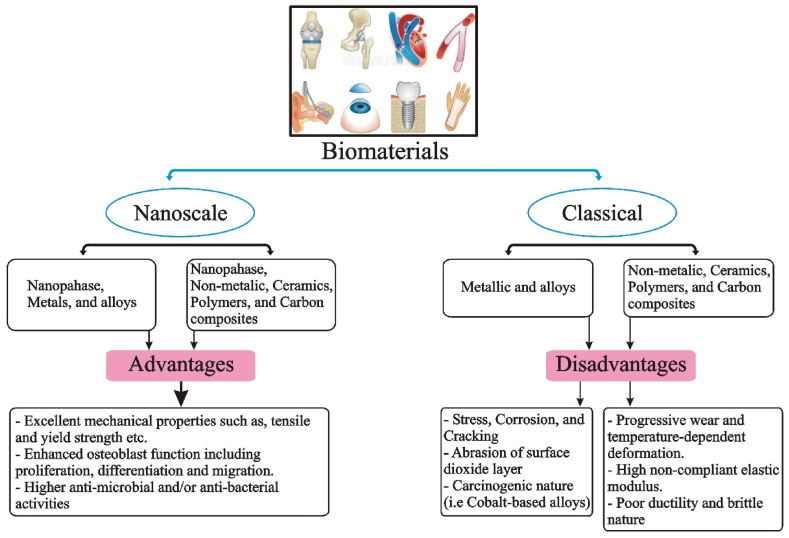
Orthopedic biomaterials are divided into two main groups: conventional and nanophase biomaterials. The former splits into (i) non-metallic biomaterials and (ii) metallic and alloys. The latter divides into (i) nanophase non-metallic biomaterials and (ii) nanophase metals (and composites). For implant uses, the traditional biomaterials face several difficulties, including progressive wear, temperature-dependent deformation, stress corrosion cracking (for 316 L stainless steel), and wear over time. Contrarily, nanomaterials have several appealing qualities, like improved functions of osteoblast and superior mechanical characteristics. (regarding tensile strength, and yield strength, etc.).

The purpose of biomaterials in orthopedic applications is to either substitute or repair the internal strength of broken bone [21]. For instance, the rigidity of the cell matrix can be changed to optimize the growth of muscle cells. Orthopedic biomaterials in the contemporary era can be divided into two main groups: nanophase biomaterials and classical biomaterials. (Fig. 2). Metals and their alloys and non-metallic elements can be used to categorize further traditional biomaterials (i.e., polymeric, carbon biomaterials, crystalline ceramics, and amorphous glasses). The limitations of traditional biomaterials for practice in orthopedic implant procedures are addressed in this section.

### 3.1 Metals and alloys

Orthopedic implants for internal fixation and weight-bearing are typically made of metals and alloys. Such implants are tightly attached to bones to ensure minimum movement between the implant and host tissue, in addition to load-carrying capabilities at the implantation site. Even though these materials are widely available, only a small number of them are successful in implant applications. For example, Magnesium is used as a load-bearing component in biodegradable orthopedic implants [22]. In the repair of bones and joints, titanium is used [23], surgical-grade stainless steel (typically 316L) is used in impermanent implants (such as fracture plates and hip nails) [24], and Orthopedic devices use metals based on cobalt (for knee, shoulders and hip) [25]. Mechanical characteristics are necessary for (i) enhancing or maintaining the fracture's structure, (ii) realigning the fragments of bone in orthopedic bioactive materials uses, and (iii) joint replacements. Metallic nanoparticles were utilized for the first time during the Industrial Revolution in the 1860s when the metal industry started to grow. Because of the uniformity of their qualities—high strength, toughness, and endurance, for example—as well as the manufacturing's simplicity and suitable biocompatibility, all of which are desirable for extending the lifespan of implants [26], in the creation of biomedical implants, metallic elements play a major role.

There are other restrictions on the use of metallic devices. Permanent implants no longer use stainless steel but rather cobalt-based metals due to the latter's superior corrosion resistance. However, *in vivo*, the emission of carcinogenic ions from cobalt-based alloys has been documented. As previously discussed, titanium and its alloys encourage the formation of a titanium oxide layer, the abrasion of which can discharge debris particles into the nearby tissues. An adverse tissue reaction and the aseptic loosening of the device over time may result from these particles. Magnesium implants have the same problem, as they constantly release hydrogen gas when they come into touch with fluids and corrode quickly. However, older people may benefit from metallic implants because of their restricted longevity (typically 20–25 years). This is a significant drawback compared to the average human lifespan. It has been noted that the durability of metallic bioactive materials used in orthopedic implant procedures (such as bone fixators) is unsatisfactorily poor after the implant's expected lifetime of 10–15 years. The risks associated with implant materials (such as corrosion behavior, metal-related, lifespan, and toxicity) prompted the research and creation of substitutes.

### 3.2 Non-metallic materials

Non-metallic materials, for example, carbon composites, crystalline ceramics, amorphous glasses, and polymers, all offer sophisticated properties for structural insertion. These materials' intrinsic biocompatibility or lackluster mechanical properties limited their use. Many people have worked hard to improve them as structural devices over the years [27]. Polymers, in contrast to metallic implants, (i) can slowly restore the functionality of tissues without the use of enzymes or catalysis [28], and (ii) over some time, polymers can transmit stress to an injured region, facilitating adequate mending of tissues [29]. In contrast to annealed stainless steel, unaided degradable polymers exhibited a 36 % higher level of deformation and 54 % more bending at the beginning. If degradable polymers are reinforced with either non-degradable carbon fiber or biodegradable synthetic fiber, their strength increases by 62 % and 15 %, respectively, when compared to unaided polymers and stainless steel, respectively.

The outstanding corrosion resistance, excellent biocompatibility, and wear resistance of ceramics have made them a compelling alternative to metallic materials for various applications. Their strong chemical stability in different physical environments and ability to withstand compression when subjected to load-bearing conditions inside the human body have further increased their appeal for such purposes. Orthopedic implants are typically made of ceramics like aluminum oxide, hydroxyapatite-based bioglass, hydroxyapatite (HA), silicon oxide, and zirconium oxide [30]. CaP ceramics are highly reactive and biocompatible, making them appealing as implant coatings. CaP porous implant coatings promote early bone development and secure implant placement. Compression stress is not easily transferred to these fabrics. The primary problem with ceramics is that, in contrast to bone, they have a much higher noncompliant elastic modulus, which can lead to either loosening or fracture of acetabular sockets. The capability of these biomaterials to stimulate bone growth and bone regeneration at the tissue-implant interface is crucial to their success. Low ductility and brittleness limit their use for fracture stabilization.

Several studies and efforts have been made to modify traditional biomaterials for use in tissue integration and bone regeneration [31]. There is still room for development in their ability to precisely direct and affect cell and tissue functions before implantation. Nanotechnology has received a lot of attention over the past decade, and many various kinds of nanomaterials have been created using physical, chemical, and even biological methods. Nanostructured extracellular matrices are readily interactable with natural tissues and organs due to their dimensional differences [32].

#### 3.2.1 Biomaterials based on ceramics

Researchers have studied ceramic-based materials for medical uses such as implant devices [33] due to their similarity to human bone tissue components like zirconia, silica, alumina, tetra calcium phosphate, tricalcium phosphate, and calcium phosphate.

There has been extensive research on ceramic nano biomaterials for various orthopedic and dental uses, particularly those based on calcium phosphate, such as β-tricalcium phosphate (β-TCP) and hydroxyapatite (HAp). The Ca/P ratio plays a crucial role in the degradation and bioactivity of calcium phosphates, which are significant in human biology. These nanomaterials can be employed in the medical field in various forms, including nanoparticles, coatings, and binders [34]. Currently, calcium phosphate-based materials are used in biomedical settings for a variety of purposes, including but not limited to drug administration, dental applications, coating of orthopedic devices [35], and bone reconstruction. Calcium orthophosphate bioceramics are used in a variety of biomedical products [36].

The biological functions of calcium phosphates and the ability to control their mechanical properties have made this family of ceramics very popular for use in a variety of biomedical uses. A lot of work is being done right now to create calcium-phosphate polymer composites, particularly HAp and TCP.

#### 3.2.2 Bioactive glasses

A distinct family of materials called bioactive glasses is well-known for its capacity to interact with biological tissues and facilitate healing processes [37]. These glasses are particularly ideal for a variety of biomedical applications because they may connect with bone and soft tissue. Their main constituents are silica (SiO2), calcium oxide (CaO), and phosphorus pentoxide (P2O5). A hydroxycarbonate apatite (HCA) layer, which is physically and chemically comparable to the mineral phase of bone, is created when the surface of bioactive glasses reacts with bodily fluids [38]. By promoting osteoconduction and perhaps inducing osteogenesis, this bioactivity can improve bone regrowth. Furthermore, therapeutic ions like silicon, calcium, and phosphorus that further aid in tissue healing and have antibacterial qualities can be created to be released from bioactive glasses [39].

#### 3.2.3 Polymer-based biomaterials

Numerous polymers have been thoroughly studied in recent years for use in bone tissue engineering. Natural and artificial polymers can be categorized as biodegradable polymeric materials. Collagen, which is a biological protein, and gelatin, which is produced from collagen, are examples of naturally occurring polymers that have shown issues like immunogenicity, instability, incompatibility, and poor biodegradability. On the other hand, most synthetic polymers, including poly(lactic-*co*-glycolic acid) (PLGA) and poly-urethanes (PURs), offer excellent applicability [40] due to their most modifiable characteristics. These biodegradable materials can be applied to tissue engineering, medication administration, dentistry applications, cardiovascular applications, orthopedic devices, and wound care. Polymers, both natural and synthetic, serve important functions in modern health. Polyesters have garnered more attention compared to other kinds of synthetic polymers. This set of polymers can be categorized into three significant classes.

Due to their advantageous properties, synthetic polymers like poly-L-lactic acid (PLLA), polymethyl methacrylate (PMMA), poly-L-lactic acid (PLGA), and polyether ether ketone (PEEK) are the mainly used polymers for medical applications. Due to its ability to integrate with host tissue despite requiring a second surgery, biocompatibility with host tissue, and hydrophobic nature, PLA could be regarded as one of the best choices for many biomedical applications [41]. Thermoplastic PLA is made from renewable materials like sugar cane and corn starch and is biodegradable. It comes in a variety of forms, such as PLLA, poly-DL-lactic acid (PDLLA), and poly-D-lactic acid (PDLA), and can be used for a variety of things, including the creation of plates, rods, screws, and pins. PLA is a polymer that crystallizes slowly and is semi-crystalline [42]. The functions of the injured bodily parts in terms of soft tissues (such as ligaments, tendons, muscles, and skin) and hard tissues (such as tendons, bones, and cartilage) determine the applications of polymers [43].

**Fig. 3:**
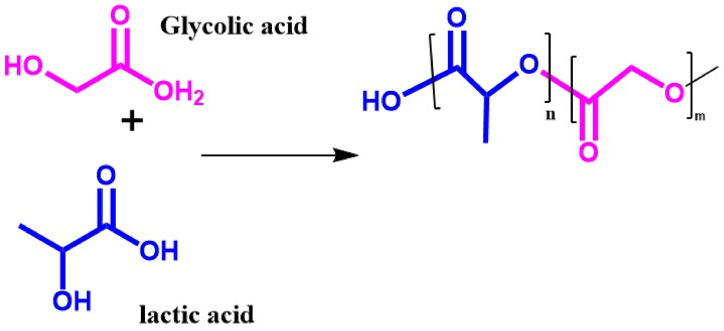
PLGA's chemical composition of its monomers and respective polymer (The numbers n and m illustrate how many times each unit is repeated). For non-load-bearing uses that demand a great degradation rate, polymer-ceramic composites like PLLA/HA may be a suitable option. Prior research indicates that the hydroxyapatite inclusion particles raise the glass transition temperature of the polymers despite affecting their crystallinity or melting point [46]. Polymer-ceramic composites, i.e., PLLA/HA, may be a viable option for uses that do not need load-bearing and necessitate a rapid rate of degradation [35]. Earlier studies [47] have demonstrated that the incorporation of HA particles elevates the glass transition temperature of the polymers, albeit it affects neither their crystallinity nor their melting point.

PLGA is another artificial biopolymer that has attracted a lot of interest. Due to its strong cell adhesion, desirable mechanical properties, controllable degradation rate, and safety, PLGA, a biodegradable polymer, has a great deal of promise for use in these applications [44]. The random ring-opening copolymerization of PLA and PGA is known as PLGA. In this way, the rate of PLGA product breakdown is adjustable by varying the proportion of these two polymers. PLGA is, therefore, favored over PGA and may be used in several biomedical applications, including cancer drug delivery systems and sutures [45]. The process of bone growth and healing has shown a significant acceleration when the implants are composed of PLGA. The molecular composition of PLGA and its monomers are depicted in Fig. 3.

PLGA (Polylactic-*co*-glycolic acid) is a well-studied and commonly utilized polymer for biodegradable medicinal applications [48].In 1996, the Food and Drug Administration (FDA) of the United States gave its clearance for medical applications [36]. This polymer is composed of monomers such as PLLA (poly L lactic acid), PDLA (poly D lactic acid), and PDLLA (poly DL lactic acid), which are non-toxic [49]. The glass transition temperature of PLA, which is roughly 55 °C, makes it brittle despite its great mechanical strength [50]. Numerous initiatives have been taken to increase these polymers' flexibility and processability. For instance, the most popular techniques for improving the elasticity of polymers are the application of plasticizers and polymer blending [51].

Previous studies have shown that the size and shape of HA particles have a significant impact on the fracture behavior of the composites. The micro- and nanoscale, as well as the spherical or plate-shaped HA particles, play a crucial role in this regard. For example, nano-HA-PLLA composites exhibit a fractured and brittle surface due to the nanoscale interactions between PLLA fibrils and primary HA particles, whereas micro-HA-PLLA composites demonstrate the highest critical release of energy rate and the most extensive surface roughness [52]. In this research, pure PLLA recorded a maximum stress of about 2.8 ± 1.2 MPa and a strain at a break of 38 ± 7 %. However, the highest stress in the micro-composite samples was five times lower, at 0.57 ± 0.2 MPa, and the strain at break was higher, at 41 ± 8 %. These findings highlight the importance of optimizing the mechanical properties of PLGA composites for their successful application in medical implants. The incorporation of HA particles particularly at the nanoscale and the blending of novel blending techniques are essential strategies to enhance the performance and durability of PLGA based nano biomaterials. The advance of composite scaffold materials is a desirable approach since it enables the combination of two or more different materials' favorable features to fulfill the host tissue's physiological and mechanical requirements. The scaffold's mechanical reinforcement can be achieved by leveraging the polymer's formability and adding controlled-volume portions of the bioactive ceramic phase. Furthermore, the generally low bioactivity of polymers can be overcome through this method.

One of the primary motivations for creating bioactive glass/polymer composite scaffolds for the engineering of bone tissue is to give the polymer matrix bioactive properties, which can be accomplished by adding bioactive coatings or inclusions. The level of substance bioactivity is affected by percentage, size, shape, and inclusion arrangement. In certain applications, the use of fibers instead of particles is preferred because higher bioactivity is favored by a greater volume fraction and a higher surface area-to-volume ratio of inclusions. To produce highly porous structures, a variety of foaming techniques are available, such as sol-gel methods [53].

## 4 Fabrication technologies

### 4.1 Solid freeform fabrication techniques (SFFT)

The SFFT techniques were used to fabricate very consistent scaffolds with completely interrelated porous networks [54]. The scaffold construction can be precisely designed by using digital data generated by imaging sources like magnetic resonance imaging and computer tomography [55]. To fabricate scaffolds with controlled micro- and microporous architectures, a combination of solid freeform (SFF) development and conventional foam scaffold manufacturing methods, such as orogen leaching, emulsion-solvent diffusion, or phase separation, can be employed. The engineering of structural tissue and multi-tissue interfaces may benefit from the use of such biomimetic internal designs.

There is no existing research or data on the SFFT-produced degradable polymer/bioactive glass composites, as far as the authors are aware. This method has only been used for compounds where the bioactive phase is composed of calcium phosphates [54]. For example, Xiong et al. [56] created PLLA/TCP composites by applying low-temperature deposition according to a layer-by-layer manufacturing technique for SFF fabrication. These composites had porosities of up to 90 % and mechanical characteristics like cancellous bone in humans (i.e., computer-driven through 3D digital models). Dioxane and TCP powder were combined with PLLA to create a slurry, which was then shaped into frozen scaffolds and freeze-dried. Both macropores (400 m in diameter) and micropores (5 m in diameter) were created by the sublimation of the fluid during the freeze-drying process. PLA scaffolds with computationally designed (500–800 m wide channels) and locally generated (50–100 m wide voids or 5–10 m length plates) pores were created by Taboas et al. [54]. Improved control over scaffold form and pore architecture, which includes interconnectivity, orientation, geometry, size, branching, and porosity, was achieved through indirect fabrication employing casting in SFF molds. This approach has the drawback of requiring more time to fabricate the scaffold than direct techniques because a temporary mold needs to be created first (Table 1).

**Table 1:** Comparison of Biomaterial production methods.

Method	Advantages	Disadvantages	Reference
**Sol-Gel Processing**	High purity	Limited to "soft" materials	[57]
	Homogeneous composition	Challenges in incorporating biomolecules	
	Control over porosity and structure	Limited to specific processing conditions	
**3D Bioprinting**	Precise control over structure and design	Limited range of viscosities for some methods	[58]
	Ability to incorporate living cells	Challenges in large-scale production	
	Potential for creating complex structures	Material restrictions (e.g., photopolymers)	
**Electrospinning**	High surface area for enhanced properties	Limited to specific polymer types	[59]
	Fine fiber diameter for biomimicry	Challenges in scaling up for mass production	
**Solid free-form fabrication (SFFT)**	High precision	Not economical	[60]
	Customizable	Restricted options for materials	
**Melt spinning**	Production rates are high	Limited to thermoplastics	[61]
	Budget-friendly	Limitation control in fabric diameter	
**Lyophilization**	Generates extremely porous structures	Time-consuming	[62]
	Maintains the biological activity	Specialized equipment is required	
**Chemical vapor deposition**	Precise coatings are produced	Toxic chemicals are produced as the process requires high temperature	[63]
	Complex shapes can be coated easily		
**Thermal processing**	Widely applicable	High energy demand with potential material degradation	[64]
	Produces durable and strong materials		

### 4.2 Leaching of particles and solvent casting

Creating biomaterial scaffolds through solvent casting involves dissolving the polymer in an organic liquid, mixing it with ceramic granules, and pouring the mixture into a 3D mold before allowing the solvent to evaporate. One of the primary advantages of this method is its convenient usage without the need for specialized equipment. However, solvent casting has several drawbacks, including shape limitations that restrict the formation of only flat sheets and tubes, the potential for toxic solvent retention within the polymer, protein denaturation, and the inclusion of additional molecules in the polymer resulting from the solvents used. Using organic solvents to cast the polymer may also reduce the effectiveness of bio inductive molecules. Detailed information regarding the processing steps may be found in the literature [65].

The solvent aggregation technique can be employed to create polymer-ceramic structures. Traditional water/oil/water emulsions are first used to create polymer nanoparticles. Then, pre-hardened microspheres, ceramic granules, solvent, and salt/sugar can be combined to create solvent-aggregated polymer-ceramic structures [66]. Based on this technique, coupled with microsphere packing and particle leaching, a 3D framework with controlled porosity is created. A typical pore morphology is produced utilizing this method. The solvent casting technique and the method have comparable benefits and drawbacks [65].

Particle leaching has not received much attention in the development of bioactive polymer-ceramic frameworks. The ability to achieve pore interlinkage at modest orogen (sucrose or salt) loadings is undoubtedly a disadvantage of this method, as numerous orogen particles will possibly stay trapped. However, this method has been used to create composites with varying and graded porosity that contain calcium phosphate inclusions [67].

### 4.3 Thermally induced phase separation (TIPS)

The TIPS method can be used to create 3D resorbable polymer scaffolds with extremely high porosities (∼97 %) to provide controlled macro- and microstructures suitable as scaffolds for tissues like teeth, bone, intestines, ligaments, tendons, muscles, and nerve [68,69] The scaffolds that are produced have widespread pore interconnectivity, anisotropic tubular morphology, and high porosity. Variations in the polymer and solvent used, quenching temperature, fraction of volume of secondary phase, and polymer concentration in solution can be used to alter the produced foams of TIPS microporosity as well as their degradation rates, bioactivity, mechanical properties, and pore morphology [68].

To achieve a uniform polymer solution, the polymer was dissolved in dimethyl carbonate for a short duration and, after that, subjected to overnight mixing. The polymer combination has the potential to be enhanced by the addition of a certain quantity of ceramic and glass powder. Once the mixture is transferred to a flask, it is sonicated. The beaker is then rapidly cooled in liquid nitrogen and stored at a temperature of −196 °C for 2 h. The frozen mixture is then placed into a cooling bath connected to a vacuum pump at −10 °C. The solvent is evaporated for 48 h at −10 °C, then for another 48 h at 0 °C, and finally dried at room temperature in a vacuum furnace until a constant weight is reached [70].

It is well-known [69] that non-porous biodegradable polylactides degrade more quickly than their porous cousins as a result of autocatalysis. This impact is caused by the fact that porous materials can make it easier for degradation products to dissolve and spread throughout the aqueous medium, which prevents autocatalysis. As determined by the hydroxyapatite formation on the composite surfaces after immersion in SBF, PDLLA/Bioglass® composites show high bioactivity [71]. Additionally, it has been demonstrated that the foams promote MG-63 cells' viability, spreading, adhesion, and migration [71].

## 5 Nanotechnology for orthopedic implants

**Fig. 4:**
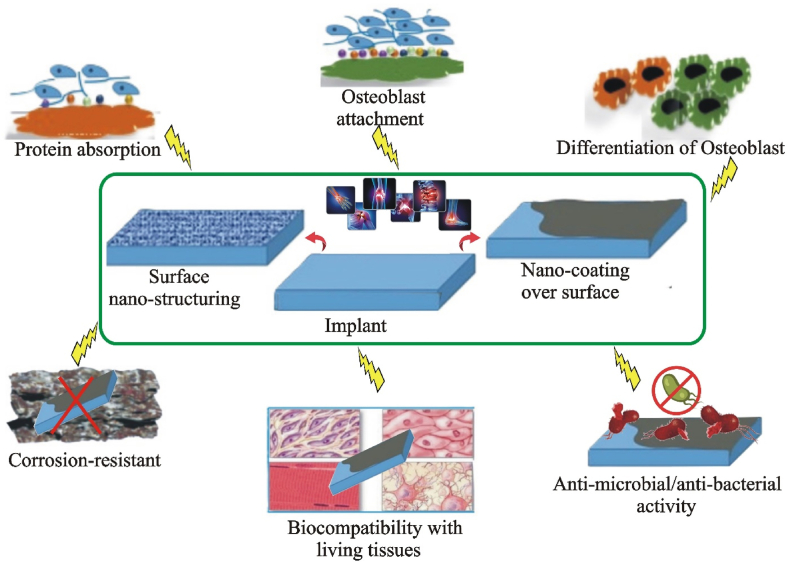
Representation of nanomaterials applications in bio-implants and salient features of nanomaterials coating over bio-implants such as bioactive nature, adaptable surface characteristics, adsorption of protein, attachment of osteoblast, and differentiation for the new bone formation.

Nanomaterials were investigated for applications of bioimplants because of their bioactive nature and adaptable surface characteristics. The nanomaterials increase physiochemical properties, roughness, effective stiffness, and surface area to improve adhesion, proliferation, protein synthesis linked to bone, and calcium mineral deposition [72]. Nanomaterials are intriguing candidates for orthopedic implants because they can replicate the extent of the individual biological bone components. To improve osteoblast functions, encourage osseointegration, and support the healing of bone-related illnesses, for instance, composites and nanostructured polymers have been extensively researched in bone tissue engineering [73]. The selection of nanostructures, such as nanorods [74,75], nanopillars [76], nanoflowers [77], nanocubes [78], nanotubes [79], quantum dots [80], and metal-organic frameworks [81] is critically essential to take into account to guarantee the functionality and dependability of the implant. Many research studies were conducted to examine the beneficial surface properties of nano-structured biomaterials, which can encourage or assist in contrast to traditional materials in (i) promoting good differentiation and migration of osteoblasts, (ii) facilitating a significant number of specific protein interactions, and (iii) improving the attachment of osteoblasts (Fig. 4) [82]. Here is a justification for each bioactivity represented in Fig. 4:

1. Protein absorption

Surface nanostructuring enhances protein adsorption, allowing proteins like fibronectin and vitronectin to adsorb onto nano-structured surfaces, facilitating cellular interactions and tissue integration [83].

2. Osteoblast attachment

Nanostructured surfaces enhance osteoblast adhesion by mimicking the extracellular matrix and increasing the local concentration of adsorbed proteins, thereby improving cell attachment [84].

3. Osteoblast differentiation

Nano-topographies can enhance bone tissue formation and implant integration by modulating cell behavior, promoting osteoblast differentiation, and influencing cytoskeletal arrangement and gene expression [85].

4. Corrosion resistant

Nano coating enhances implant corrosion resistance by providing protective barriers and enhancing stability, making them less permeable and less corrosive to the underlying material [86].

5. Biocompatibility with living tissues

Nano coatings and nano-structured surfaces mimic the biological environment, reducing immune responses and improving compatibility with the living tissues thereby reducing rejection or inflammatory responses [87].

6. Anti-microbial/antibacterial activity

Nano materials can be functionalized with antimicrobial agents like silver nano particles, which can inhibit bacterial adhesion and colonization by creating physical barriers or causing unfavorable surface energies [88].

### 5.1 Implantable nanomaterials

The advancement of nanotechnology has led to the emergence of various nanophase materials, such as biomaterials, metals, ceramics, and polymers, that possess unique surface properties due to their grain size of less than 100 nm. These materials demonstrate the potential to promote osseointegration and stimulate bone growth [89]. Researchers have observed a 20-fold increase in cell proliferation after reducing the titanium grain size from 4.5 μm to 200 nm through equal channel angular pressing [90]. Nanophase biomaterials typically comprise multiple grain boundaries due to their distinct atomic structure, which imparts great hardness and/or strength and low flexibility and/or brittleness [91]. However, their brittleness can pose significant challenges in certain structural applications. This brittle behavior can be attributed to various factors, such as their compact production and fundamental makeup [92].

The safety of carbonaceous materials has limited their exploration for orthopedic applications. Thus, very little research has been done to determine the best ways to synthesize nanofibers (CNTs/CNFs) and carbon nanotubes for orthopedic prosthesis devices [93]. However, CNTs are a hopeful material for implants due to their structural and mechanical qualities. Additionally, it has been discovered that CNT-based composites have less toxic impacts than asbestos [94]. It has been suggested that adding CNTs to polystyrene (PS) matrixes, polycarbonate-urethane (PCU), and polycaprolactone (PCL) will improve the mechanical characteristics of the composite scaffolds (in terms of tension and compressive moduli) [95]. Nanocomposites are often considered superior to other materials due to the combined benefits provided by each component, leading to enhancements in their mechanical, physical, and chemical characteristics.

## 6 Recent developments in biomaterials for orthopedics

The most important goal of research in orthopedic implant applications and tissue engineering is the creation of stimulus-responsive biomaterials with adaptable properties. Traditional biomaterials, which usually consist of just one component, have several drawbacks, including inadequate control over biophysical and biochemical properties, which tend to restrict their usefulness in biomedical applications. Significant advancements have been noted recently with new trends like 3D implants [96], porous structures [97], engineered biomaterials, and smart biomaterials [98] from natural sources [99]. Combining the qualities of various types of natural polymers having high biocompatibility (such as keratin, collagen, chitosan, elastin, and silk) along with synthetic polymers with favorable mechanical characteristics (such as Teflon, epoxy, polyester, and polyethylene), has led to the development of new biomaterials [100]. Biomaterials are designed to imitate living tissues for applications such as gene therapy, cell-based transplantation, and tissue engineering. Recent advancements in fabrication technologies and natural biomaterials have enabled the creation of a new type of smart nanomaterials, including nanoporous anodized alumina (NAA), which can be used for these purposes [101].

Nanopores are attractive for various purposes like gene therapy, cell-based transplantation, and tissue engineering due to their large surface area, which allows for high surface loading, and their nano-confined volumes that can control protein breakdown rates [102]. In the context of attaching bioactive molecules to implants, it is advisable to refrain from utilizing intermediary linkers such as phosphonates and xylenes in nanopore-based systems due to their elevated surface area [103,104]. Moreover, nanopores can be utilized as bioactive coatings on diverse biomaterial surfaces to improve cell adhesion and protein adsorption [105]. Additionally, studies have demonstrated that porous metals with structures suitable for orthopedic applications have the potential to replace broken bones with ones that possess similar characteristics to natural bones [106]. This article provides an in-depth analysis of the potential application of 3D implants and smart biomaterials in this area.

### 6.1 Advancements in 3D orthopedic implants

The use of 3D implants is a promising trend for creating desired structures with target material properties which is superior to monolithic structures. Recent advances in 3D bioprinting techniques have made it possible to produce intricate organs with vascular tissues, skin, and bone, as well as scaffolds for stem cell differentiation. Successful transplantation of cartilage tissue in humans has been achieved using this approach [107]. Selecting the appropriate biomaterials (such as tricalcium phosphate, HA, and polyphosphate with Mgo and SrO doping) and ensuring shape specificity are critical factors to consider when attaching biomolecules to facilitate cell differentiation, proliferation and adhesion in 3D implants [108]. There are numerous possibilities to create bioimplants with finer structures and better processing resolution. Custom coatings that are meant to alter the osseointegration of bioimplants can be produced using 3D printing technology, such as 3D-printed trabecular titanium [109].

Regarding applications in biomaterials, various 3D printing technologies exist. These include robotic-assisted deposition/robocasting [110], direct ink writing (3D plotting) [111], fused deposition modeling [112], laser-assisted bioprinting [113], selective laser sintering [114], and stereolithography [115]. All the technologies, however, have advantages and disadvantages. For example, 3D plotting/direct ink writing, despite having a restricted range of viscosities and complicated processing, allows for easy integration of both drugs and biomolecules (such as living cells and proteins) [116]. Similarly, stereolithography creates simple and complex designs layer by layer but is only suitable for photopolymers. The most widely used methods for industrial use with metallic materials are electron beam- or laser-based 3D printing, which have been improved [117]. To modify the osseointegration of metallic implants, Nasiri et al. [188] produced a 3D nanocrystalline HA shape with high porosity and a micro-nano structural hierarchy. When coated with a porous nanoparticle network of crystalline HA, titania implants may have better osteoconduction and osseointegration at the bone-implant interface [118].

Complex tissues pose many difficulties for bioprinting that must be overcome before they can be used clinically, including bioprinting stresses, the potential for proliferation, and appropriate functionality [119]. The ability of 3D bioprinted tissues made up of various cell types to vascularize, endure, and proliferation has been proven [120]. Microwave sintering was used by Tarafder et al. [121] to create 3D-linked macro-pore tricalcium phosphate (TCP) scaffolds. These 3D scaffolds hold a lot of promise for tissue regeneration and healing. Microwave sintering has several advantages over conventional sintering, including faster, more uniform heating of a sample, improved mechanical properties (e.g., 10.9 MPa and 56.62 MPa compressive strength for scaffolds with 500 μm pores sintered in microwave and typical furnaces, respectively), higher densification, and organized grain growth without the development of cracks. The manufacture of 3D bioactive glass-ceramic scaffolds with high compressive and flexural strength has been made possible using direct ink writing methods, which utilize strontium-doped hardstone-gahnite. These scaffolds, which have a compressive strength of 110 MPa and a flexural strength of 30 MPa, are comparable to cortical bone [122]. Biopolymers found in nature, such as alginate and chitosan, have also been used for 3D bioprinting, in addition to ceramics, due to their unique qualities, including their similar molecular structure, inherent biocompatibility and high water content that is comparable to the extracellular matrix [123,124].

By mixing the right fillers into the matrix powder, it is possible to improve the biological characteristics of implants (such as their mechanical strength, regulated bioactivity, and biodegradability) [125]. To create 3D scaffolds for biological applications, Chakravarty et al. [126] used a spark plasma sintering method. Graphene has been identified as a highly potential material for implants due to its outstanding mechanical properties, thermal stability, biocompatibility, very high porosity levels ranging from 35 to 50 %, and extremely high cell viability. Recent studies have tended to concentrate on 3D-printed scaffolds that, using a biomimetic plywood construction, resemble bone tissue structurally. This plywood construction can assist in reducing some of the defects found in 3D scaffolds, such as plastic deformation, fracture propagation, and asymmetric buckling [127].

The major concerns regarding the extensive use of 3D-printed bioimplants are regulatory. For instance, it is crucial to achieve desired material properties with the necessary structure, to ensure that the fabrication process takes place in a sterile environment, and to address the interactions between drugs and materials during the 3D printing process. These are critical problems that need to be resolved through significant process improvements before being implemented in clinical settings.

### 6.2 Smart orthopedic biomaterials

The creation of smart biomaterials has become a subject of intense study. In this context, "smart" refers to the nature of interactions between a biomaterial and its immediate biological environment, including cells and tissues. Smart biomaterials stand out from conventional biomaterials due to their instructive/inductive (or stimulating/triggering) impacts on nearby cells and tissues. In addition to directly contacting host cells to provide various physical and chemical cues, smart biomaterials can also be closely associated with host constituents for extended periods [128]. However, achieving the necessary level of complexity to accurately replicate the extracellular matrix of natural tissues for the correct cellular attachment stimulation and cell proliferation is the primary challenge in developing smart biomaterials.

**Fig. 5:**
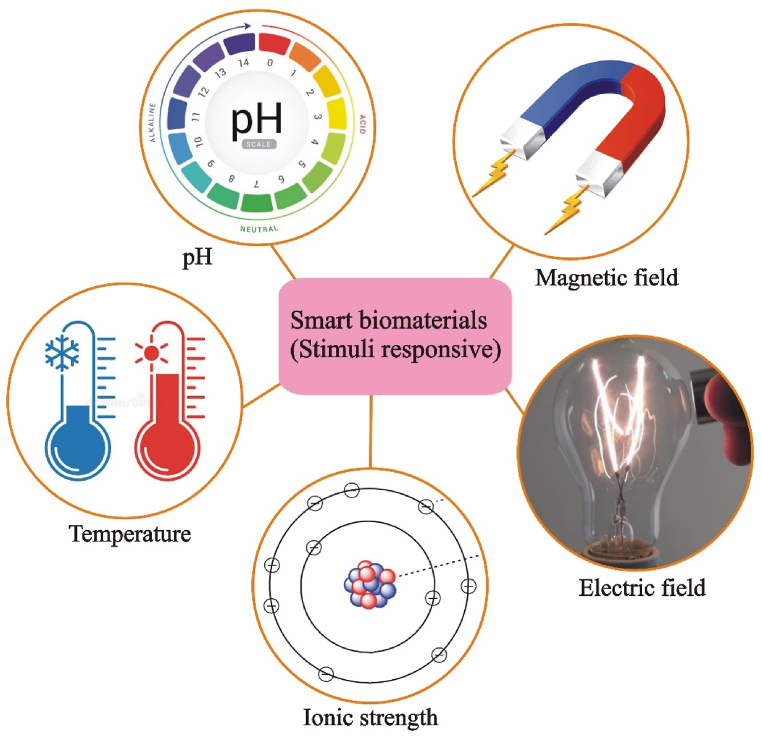
Behavior of intelligent orthopedic polymers in response to stimuli.

Recent investigations have focused on generating composite biomaterials that possess intelligent characteristics like imitating the physical and chemical properties of native tissues, their inductive/instructive influence on cells, controlled/sustainable transportation of biofactors, and their delivery that reacts to stimuli such as temperature and pH, as shown in Fig. 5. These materials have a wide range of applications, from soft tissues to hard tissues such as bones and teeth [129]. A smart biomaterial implant can play an active role in the regeneration of injured tissues by responding appropriately to stimuli in the biological environment. In creating composite smart biomaterials, the preferred fundamental building block has been synthetic hybrid block polymers and peptides [130]. The traditional methods of bone replacement (such as autografts and allografts) suffer from a lack of supply, donor site morbidity, and infection risk [131]. Recent research has focused on scaffold-based bone implants, which use a variety of biomaterials, primarily ceramics and aliphatic polyesters, to provide favorable stability and enable the customized construction of implants [132]. Comesaña et al. [133] utilized a laser-assisted production technique to produce personalized bone implants with an outer layer of bioactive glass (that is more easily broken down) and an inner core modified with CaP (which has a moderate degradation rate). Such implants have the potential to incorporate properties like angiogenic, antibacterial, or antiresorptive activity and are frequently used for minimal load-bearing bone restoration. Hyperbranched polyurethane (HBPU)/Fe_3_O_4_ nanocomposites made with sunflower oil were created by Das et al. [134] using an in situ polymerization method. In comparison to their original system, the polymer matrix with Fe_3_O_4_ incorporated provided improved antibacterial activity, biodegradability, biocompatibility, and thermomechanical properties. Bio-based polymeric nanocomposites are especially promising and appealing for implant applications due to their clever magnetically controlled behavior [135].

Nanogels, microgels, and smart hydrogels are some of the materials that respond to stimuli the quickest at the moment, whether it be a change in temperature, ionic strength, pH, or magnetic or electric fields [136]. These materials are more hopeful as smart biomaterials for orthopedic implants because they are biocompatible and biodegradable [137]. To induce the formation of minerals in MG63 osteoblasts and typical human osteoblasts without resorting to external osteogenic factors like calcium mineral deposition, a scaffold made of gelatine methacryloyl (GelMA) was placed on top of a titanium implant surface [138]. To develop versatile structures capable of supporting mechanical stresses and interacting with electroactive tissues, hydrogels have the potential to be enhanced through the incorporation of nanomaterials such as graphene, CNTs, clay-based platelets, and ceramic nanoparticles [139]. Further improvements in regulating cell activity through the matrix are necessary to address interactions between cells and biomaterials.

### 6.3 Synthetic biomimetic materials allow cellular remodeling and tissue regeneration *in vivo*

Biomimetic materials made of synthetic materials have been developed to act as temporary matrices for tissue regeneration *in vivo*. The idea behind this is that fibrin acts as a temporary matrix in tissue repair. Synthetic biomimetic materials must possess certain properties to promote regeneration *in vivo*, such as ligands for cell adhesion, a mechanism for relatively rapid and localized matrix dissolution that is ideally synchronized with cell invasion, and the ability to deliver morphogenetic signals to attract endogenous progenitor cells and induce their differentiation. Using this concept, bioactive materials were created with integrin-binding adhesion ligands and bone-inducing growth factor BMP-2 that were sensitive to MMPs or plasmin. When these materials were implanted in bone defects, the matrix transformed completely into bone. This concept of a matrix that is responsive to cells and adhesive to cells could be applied to other areas of regeneration [99].

### 6.4 The biological performance of bioactive material in bone allografts

The use of recombinant growth factors derived from humans, derivatives of enamel matrix, various platelet-rich preparations, and either the patient's bone or cultured bone-forming cells from an external source are all capable of enhancing the biological function of bone grafts that are derived from another person [140]. Alternately, demineralized bone matrix (DBM)—a naturally occurring polymer—can be produced by treating mineralized bone allografts with hydrochloric acid (0.5–0.6 M) or a 1:1 formic acid-citric acid solution to dissolve inorganic elements [141]. Although DBM is an acellular organic matrix, it has excellent osteoinductive and osteoconductive performance and resembles the microstructure of native bone [142]. DBM contains the main proteins related to bone, such as growth factors that induce bone growth and adhesion ligands. These proteins can aid in the formation of new bone [143]. However, since DBM lacks structural support, it is mainly used in areas with stable structures, such as locations with bony defects.

Recent studies suggest that it might be feasible to develop a soluble form of ECM materials or DBM products that can be induced to form a hydrogel scaffold, even though current clinical DBM delivery typically involves incorporating DBM particles in a carrier liquid [144]. These bone matrix-based materials have unique structural, mechanical, and biological characteristics and can quickly revascularize and serve as suitable carriers for autologous bone marrow for medical applications. The graft typically does not cause a significant local foreign-body immunogenic response and supports cell attachment and growth because the antigenic surface structure of the graft is destroyed during demineralization [145]. The recent discovery of the ability of DBM to be remineralized using the alternating solution immersion (ASI) method enables the production of allografts that are mechanically sturdy, robust, and biocompatible, thereby facilitating tissue engineering and clinical applications [146]. The various proteins and growth factors present in the mineral component of these products, as well as the demineralization process that renders these factors available in the host environment, may be responsible for the biological activity of these products.

Recent scientific investigations have made notable advancements in comprehending the efficacy of biomaterials in the field of orthopedics, achieved through rigorous examination in laboratory settings as well as live organism experiments. *In vitro* investigations have been significant in revealing the cellular responses and interactions with biomaterials. An exemplification of this can be observed in research conducted by Tsakiris et al., whereby a comprehensive series of *in vitro* experiments were undertaken to investigate. The study employed Biomaterial inside a simulated physiological setting to imitate the conditions of interest [147].

Furthermore, the utilization of *in vivo* investigations has yielded significant findings regarding the practical applications of biomaterials. Another study showed the effectiveness of Biomaterial in stimulating bone regeneration in animal models. The findings of this research provide encouraging outcomes that help bridge the divide between laboratory experiments and potential clinical implementations [58]. The discoveries mentioned above highlight the significant impact that biomaterials can have in the field of orthopedics, extending beyond theoretical concepts to concrete results in controlled laboratory environments and living animals.

## 7 *In vitro* and *in vivo* characterization

For bioactive ceramics as well as biodegradable polymers alone, there are numerous *in vitro* and *in vivo* studies available; however, *in vitro,* research for polymer/ceramic mixtures has only recently begun [148]. Few mixed systems have been investigated *in vivo* up until now. More investigation is also necessary to decide whether the investigated bioactive composite substrates are appropriate for use in soft tissue engineering procedures. Additional studies on the effects of the bioactive phase and the dissolving byproducts of *in vivo* new tissue development on vascularization should be included in this study.

## 8 Challenges and opportunities

### 8.1 Incorporation of biomolecules

Surface changes, such as plasma treatment or protein adsorption, can be used to provide additional signals about cell attachment and reactivity [149]. Composites made of bioactive glasses and biodegradable polymers, or hydroxyapatite inserts have begun to be investigated for the potential to contain growth factors. It has been demonstrated that integrins, laminin, and RGD proteins are necessary for cell adhesion to material surfaces [150]. Along with encouraging cell attachment and growth, the immobilization of these proteins ought to improve the wettability of hydrophobic polymers like PDLLA. As shown for ceramic surfaces [151] and polymer surfaces, various protein immobilization methods can be used to regulate protein adhesion and release kinetics. In vivo experiments have demonstrated the osteoinductive properties of specific growth factors. As discussed, potential growth factors include transforming growth factor beta, bone morphogenetic proteins (BMPs), insulin-like growth factor, and VEGF. These growth factors might be immobilised on the scaffold surface, which would hasten bone healing and speed up patient rehabilitation.

The fabrication of scaffolds is complicated by the incorporation of biomolecules, which cannot be processed at high temperatures (470 °C) or under highly aggressive chemical circumstances. Sol-gel processing is one method of processing "soft" materials that may be used to integrate biomolecules into the construction of scaffolds. However, to the authors' understanding, bioactive organic/inorganic hybrids derived from sol-gel have not yet been shaped into very closely linked porous structures, which is necessary for using these composites as scaffolds. Clarifying the local effects of growth factors on the cell as well as tissue systems, involving long-term effects, is another related problem.

### 8.2 Commercial challenges

Before any new biomedical invention is brought to market, it is important to consider its effects from a perspective that takes into account its value. These effects can be categorized into three groups: (i) the effects on the quality of treatment in comparison to existing treatment options, such as pain reduction, increased longevity of implants, and reduced morbidity; (ii) the effects on the value of treatment (related to treatment quality); and (iii) the effects on the costs of treatment compared to those of currently available treatment options [152]. The market development of bioimplants is being hampered by some factors, such as their high cost and unfavorable reimbursement policies. Nanostructures' distinctive biological, chemical, and physical characteristics change the way devices work and how reliable they are. The difficulty lies in accurately simulating living bone structure.

The development of coatings that dissolve at a rate like bone apposition is necessary for coating approaches to create direct bone contact on the nanosurface of implants. Beyond these restrictions, thorough case studies are needed for orthopedic devices based on nanomaterials. The importance of nanophase materials for implant uses to reinforce the implant bonding with its neighboring bones has been made clear by substantial research in general [153]. It is still unknown how engineered nanoparticles are exposed to the air and water. It is important to correctly assess the impact of nanomaterials on people and animals after exposure to a contaminated environment [154]. Before these implants are approved for clinical use, careful consideration must be given to their creation, implantation, and wear patterns to assess any possible health risks.

## 9 Summary of current status and future trends

The biodegradable and synthetic polymer/inorganic bioactive phase composites discussed in this article are highly attractive as scaffolds for tissue engineering due to their bioactive properties, ability to be shaped, and adaptable biodegradation kinetics. The integration of inorganic bioactive phases into porous and interconnected 3D polymer networks has been achieved through the modification and expansion of traditional materials manufacturing methods.

The current challenge in tissue engineering, from a materials science perspective, is to design and construct repeatable bioactive and bioresorbable 3D scaffolds with pore structure and tailored porosity, which can maintain their integrity and structure for expected periods, even under load-bearing conditions. According to this study, man-made composite scaffolds still have mechanical integrity that is at least a single order of magnitude inferior to cortical or cancellous bone. The ability to replace larger sections of fractured bone tissue than is currently feasible may be made possible by attaining the mechanical characteristics of bone.

It is promising and presently the subject of extensive research to incorporate biomolecules, such as growth factors, to speed up local bone healing. However, because biomolecules are delicate to high temperatures and harsh chemical conditions, incorporating them during scaffold manufacturing is not straightforward. Immobilizing growth factors and proteins after processing by functionalizing the scaffold's surface is a hopeful approach. For the composite scaffolds discussed in this article, there are hardly any *in vitro* and, more specifically, *in vivo* studies available. However, *in vivo* and *in vitro* studies are unavoidable to address clinical applications, and it is crucial to conduct more research in biological systems. Hard tissue healing with biomaterials began with bioinert techniques, which required the production and application of bioinert materials. The bulk of irreversible bioimplants, like hip replacements, are made of these materials in today's therapeutic environments. The next phase of biomaterials research focused on the bone-bonding properties of bioactive glassware and ceramics [155]. This was soon followed by the development of biodegradable scaffold components for bone tissue engineering that can cause specific cellular responses at the molecular level [156].

The bioactive, degradable, and incorporation-possible properties of the second and third periods of biomaterials are combined in the composite scaffolds discussed in this paper. The intricacy of the biological components that the developed biomaterials have been able to handle has increased over time, starting at the level of ion interactions and moving up to the inclusion of growth factors. By engineering both bioactivity and biodegradability, biomaterials have evolved from solely synthetic materials to material/biologic hybrids. The genesis of biomaterials appears to come from the addition of stem cells, even though the focus of current research is still mainly on the interplay of stromal cells with biomaterials. As shown by Levenberg et al., in 2003, scaffolds seeded with stem cells enable local cell function modification through the differentiation of stem cells. With the help of this novel technique, the scaffold surface can imitate intricate regional biological processes, which could eventually result in the growth of organs and tissues both *in vitro* and *in vivo*. The interaction between stem cells and scaffolds, which affects growth factor absorption and cell adhesion, is currently the focus of research [157]. Our opinion is that the utilization of engineered composite scaffolds created by combining biodegradable polymer matrices and bioactive inorganic phases, as explored in this study, will be essential and potentially the preferred "scaffolds of choice" when combined with stem cell seeding (Table 2).

**Table 2:** Summary of recent use of bioactive materials in bone implants and orthopedic applications.

Study (Year)	Biomaterial Type	Biomaterial Class	Application	Key Findings
**Ielo et al., 2022** [158]	Nanocomposite	Hydroxyapatite-based	Orthopedic implants	Improved osseointegration and mechanical properties.
**Elumalai et al., 2023** [159]	Bioactive polymers	Synthetic Polymers	Bone tissue engineering	Enhanced cell adhesion and proliferation.
**Amirtharaj et al., 2022** [160]	Nanostructured coatings	Inorganic Coatings	Joint replacements	Reduced wear and increased implant longevity.
**Zhang et al., 2022** [161]	Nanostructured sTiO_2_ coatings	Titanium dioxide (TiO2)	Knee implants	Enhanced antibacterial properties and osteointegration.
**Ortega et al., 2020** [162]	Bioglass-ceramic composites	Bioglass	Spinal fusion	Improved fusion rates and biocompatibility.
**Liu et al., 2022** [163]	Injectable cements	Calcium Phosphate	Fracture repair	Accelerated bone healing with controlled drug release.
**Nitti et al., 2021** [164]	Composite scaffolds	Collagen-hydroxyapatite	Bone tissue engineering	Mimicked natural bone structure, promoting cell differentiation.
**Fang et al., 2023** [165]	Nanoparticles	Mesoporous Silica	Drug delivery in joint implants	Controlled release of anti-inflammatory drugs for arthritis.
**Sheng et al., 2022** [166]	3D-printed alloys	Porous titanium	Hip implants	Improved biomechanical compatibility and reduced stress shielding.
**Garcia et al., 2021; Desai et al., 2023** [57,167]	Hydrogels	Chitosan-based	Cartilage regeneration	Enhanced chondrogenesis and integration with native tissue.

## 10 Conclusion

Controlling the chemistry and topography of bioimplant surfaces through nanotechnology not only enhances our understanding of biological interactions but also allows for the creation of novel implant nano surfaces with predictable tissue or organ interactive properties. Nanotechnology in biomaterials, particularly in Zr-doped hydroxyapatite nanorods offers enhanced bioactivity, improved compatibility with biological systems, and superior mechanical properties potentially revolutionizing medical applications. Advancements in polymer matrix nanocomposites show potential for customized, functionalized materials for bone tissue engineering, combining polymers and nanomaterials for improved mechanical strength, bioactivity, and controlled degradation rates. Hydroxyapetite-based nanocomposites are being optimized for medical applications, and drug delivery with doping and surface modifications enhancing their properties. In this synthesis of existing research, we have aimed to go beyond the mere cataloging of previous articles, offering our unique insights and perspectives on the implications and potential of nanophase biomaterials in orthopedic implants. Our critical examination of the literature underscores the favorable characteristics that nanophase biomaterials bring to orthopedic applications, from improved bone ingrowth to enhanced tissue regeneration and optimized osteoblast functions. Notably, we emphasize the potential of implant surfaces with functional nanostructured or nanocoated surfaces to leverage nanoparticles effectively within implants.

Looking forward, we emphasize the importance of developing advanced design methodologies that seamlessly integrate the benefits of nanomaterials with cutting-edge fabrication technologies. This, we believe, is crucial for the continued evolution of nanophase biomaterials in orthopedics. While we acknowledge the promising future these materials hold, we also advocate for comprehensive investigations into the potential health hazards arising from cell-nanophase biomaterial interactions before the clinical or commercial application of orthopedic implants driven by nanotechnology.

In conclusion, our article goes beyond a mere compilation of existing literature; it offers a unique synthesis of viewpoints, critical discussions, and prospects in the realm of nanophase biomaterials for orthopedic implants. We hope this revised conclusion addresses concerns about the depth of discussion and provides a more balanced and insightful perspective.

## 11 Method of literature search

To ensure a comprehensive review of the existing literature on bioactive materials in orthopedics, we utilized multiple databases, including PubMed, Scopus, Web of Science, and Google Scholar. Our search strategy involved the use of specific keywords such as "bioactive materials," "orthopedic therapies," "biodegradable polymers," "bone regeneration," "nanomaterials in orthopedics," and "biocompatibility." We employed Boolean operators to refine our searches, using combinations like "bioactive materials AND orthopedic implants" and "nanomaterials OR biodegradable polymers AND bone regeneration." Filters were set to include only articles published in the last ten years to ensure the inclusion of recent advancements. Additionally, we reviewed the references of key articles to identify further relevant studies. Our inclusion criteria focused on studies that addressed the development, application, and evaluation of bioactive materials in orthopedic therapies, emphasizing recent advancements and clinical outcomes. We excluded articles that did not directly relate to our topic or lacked scientific rigor. Data extraction involved noting study objectives, methodologies, key findings, and conclusions, along with significant trends or recurring themes. This structured approach enabled us to synthesize the gathered data, providing a comprehensive overview of the current landscape of bioactive materials in orthopedics and highlighting their diverse applications and effectiveness.

## Ethical approval and consent to participate

Not Applicable. This is a review paper and does not involve direct research on humans or animals.

## Consent for publication

“Not applicable” as this manuscript does not contain data from any person.

## Funding

This work was supported by Public Technology Applied Research Projects of Zhejiang Province (LGF22H060023 to WQL), 10.13039/501100017531Medical and Health Research Project of Zhejiang Province (2023KY1303 to HGL, 2022ZB382 to WQL), Traditional Chinese Medicine Science and Technology Projects of Zhejiang Province (2022ZB380 to JYZ, 2022ZB382 to WQL), Science and Technology Project of Zhoushan (2022C31034 to CZ, 2022C31022 to XGH, 2023C31019 to HJZ), Research Fund Projects of The Affiliated Hospital of Zhejiang Chinese Medicine University (2021FSYYZY45 to WQL).

## Data availability statement

Not Applicable. This is a review article, and all relevant information is provided in the article.

## CRediT authorship contribution statement

**Wenqing Liang:** Writing – review & editing, Writing – original draft, Supervision, Project administration, Funding acquisition, Conceptualization. **Chao Zhou:** Writing – original draft. **Juqin Bai:** Writing – original draft. **Hongwei Zhang:** Conceptualization. **Hengguo Long:** Writing – original draft. **Bo Jiang:** Writing – original draft, Data curation. **Jiangwei Wang:** Writing – review & editing. **Xiaogang Huang:** Writing – review & editing. **Hengjian Zhang:** Writing – review & editing, Data curation. **Jiayi Zhao:** Writing – review & editing, Supervision, Conceptualization.

## Declaration of competing interest

The authors declare that they have no known competing financial interests or personal relationships that could have appeared to influence the work reported in this paper.
